# An “Electronic Fluorescent Pictograph” Browser for Exploring and Analyzing Large-Scale Biological Data Sets

**DOI:** 10.1371/journal.pone.0000718

**Published:** 2007-08-08

**Authors:** Debbie Winter, Ben Vinegar, Hardeep Nahal, Ron Ammar, Greg V. Wilson, Nicholas J. Provart

**Affiliations:** 1 Department of Cell and Systems Biology, University of Toronto, Toronto, Ontario, Canada; 2 Department of Computer Science, University of Toronto, Toronto, Ontario, Canada; Purdue University, United States of America

## Abstract

**Background:**

The exploration of microarray data and data from other high-throughput projects for hypothesis generation has become a vital aspect of post-genomic research. For the non-bioinformatics specialist, however, many of the currently available tools provide overwhelming amounts of data that are presented in a non-intuitive way.

**Methodology/Principal Findings:**

In order to facilitate the interpretation and analysis of microarray data and data from other large-scale data sets, we have developed a tool, which we have dubbed the electronic Fluorescent Pictograph – or eFP – Browser, available at http://www.bar.utoronto.ca/, for exploring microarray and other data for hypothesis generation. This eFP Browser engine paints data from large-scale data sets onto pictographic representations of the experimental samples used to generate the data sets. We give examples of using the tool to present Arabidopsis gene expression data from the AtGenExpress Consortium (**Arabidopsis eFP Browser**), data for subcellular localization of Arabidopsis proteins (**Cell eFP Browser**), and mouse tissue atlas microarray data (**Mouse eFP Browser**).

**Conclusions/Significance:**

The eFP Browser software is easily adaptable to microarray or other large-scale data sets from any organism and thus should prove useful to a wide community for visualizing and interpreting these data sets for hypothesis generation.

## Introduction

With the prevalence of large-scale data sets as a resource for biological research, tools for collecting and examining microarray and other high-throughput results are becoming increasingly significant. Currently, several databases of Arabidopsis gene expression data are accessible, including NASCArrays [1], GEO [2], SMD [3] and ArrayExpress [4]. Among the various portals for analyzing microarray data that have been developed are TAIR [5], [6], AraCyc [7], MAPMAN [8], GENEVESTIGATOR [9], and several tools of the Bio-Array Resource [10]. In addition, a database of predicted and documented subcellular localizations for most Arabidopsis proteins has been published – SUBA [11]. For mouse, microarray data forming a “tissue atlas” have been generated [12]. Such data sets have been or are in the process of being generated for human and several model organisms. The electronic Fluorescent Pictograph (eFP) Browser was developed to aid in further interpretation of gene expression data and data from other large-scale data sets. As an example of its utility, we have set up this tool as the **Arabidopsis eFP Browser** for exploring Arabidopsis microarray data to permit intuitive visualization of gene expression data across approximately 22,000 genes from *Arabidopsis thaliana*, as represented on the ATH1 GeneChip from Affymetrix. In addition, we also provide examples of how we have used it to create a **Cell eFP Browser** for displaying protein subcellular localization data and a **Mouse eFP Browser** for displaying gene expression data from a mouse tissue atlas.

In the case of the **Arabidopsis eFP Browser**, the expression data displayed include many of the results from the AtGenExpress initiative, as well as a tissue-specific collection, mirrored in the Bio-Array Resource [10] for quicker access. The user is presented with idealized images of Arabidopsis in the context of the chosen series. The user establishes the AGI ID (Arabidopsis Genome Initiative identifier) of a particular gene and the interpretative mode – absolute, relative, or compare. Upon submission, the plant tissues are coloured according to the expression level of the gene of interest in a particular tissue under a particular treatment. The tool is intended as a quick and easy means of identifying significant tissues and is particularly useful when exploring gene families to facilitate hypothesis generation. It is our goal to make this tool into a community resource whereby researchers from around the world can upload both data sets and diagrammatic representations of the experiment in question, or add it to their own databases as a free-standing tool. Users of the resource will then be able to explore high-throughput experiments by examining compact representations of the experiments overlaid with data. To demonstrate the value of the **Arabidopsis eFP Browser** in practical genomic applications, we offer examples of genes whose expression patterns have been reported in the literature, and also provide examples of displaying other large-scale data sets – in the one case the **Cell eFP Browser** to display subcellular localization data for Arabidopsis proteins [11], and in the other case, the **Mouse eFP Browser** to display gene expression data from a mouse tissue atlas [12].

## Results and Discussion

**Figure 1:**
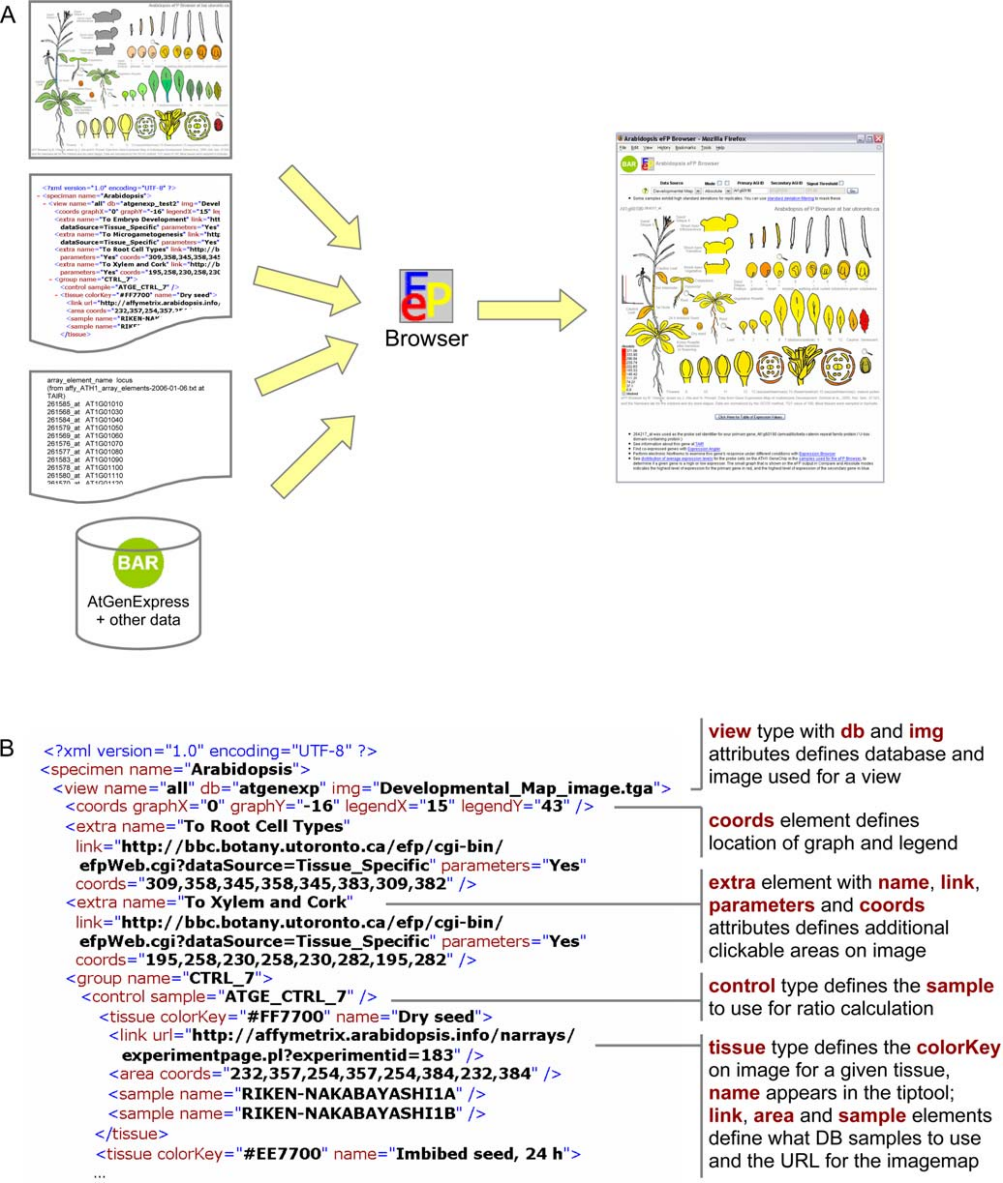
Representation of eFP Browser implementation. A. Schematic of browser inputs showing from top to bottom on the left the input image that forms the basis for the output, the XML control file, the identifier to probe set lookup, and the expression database. These are used by the eFP Browser to generate the output image upon user input. B. Snippet of the XML control file with significant tags highlighted.

We provide specific examples of using the **Arabidopsis eFP Browser** for exploring large-scale microarray data sets. The user chooses the Data Source from a list. The eFP Browser engine responds immediately by changing its display to the .png image file with the corresponding name. After the user chooses the desired mode, the AGI ID fields must be filled: the primary AGI ID field is required for all modes, as is the secondary AGI ID field in the Compare mode. When the form is submitted, the Browser engine first converts the AGI ID or IDs to Affymetrix ATH1 GeneChip probe set identifier or identifiers via a lookup table, a copy of which was obtained from TAIR at www.arabidopsis.org (a probe set identifier or identifiers may also be entered – in this case no lookup takes place, as is currently the case with the **Cell eFP** and **Mouse eFP Browsers**). Then, the eFP Browser engine parses the XML file and recovers the tissues' colour keys and sample identification for all replicates. Utilizing a MySQL command, the probe set and sample ID are used to retrieve the expression data mirrored in the Bio-Array Resource [10] for each tissue. The appropriate colour of a tissue is determined by evaluating the ratio of the averaged replicates to the positive or negative maximum and converting it to the equivalent place on the colour scale: yellow to red for positive numbers and yellow to blue for negative. We chose these colours for maximum contrast on computer screens. For each tissue, the engine loops through every pixel of the image and replaces all instances of the tissue's colour key with the calculated colour using the replaceFill function we introduced to the Python Imaging Library (PIL, www.python.org). The final output with a colour legend appended is saved as a temporary image file which is returned to the user's browser, along with other information in HTML format. This process is pictured in Figure 1.

### Features

**Figure 2:**
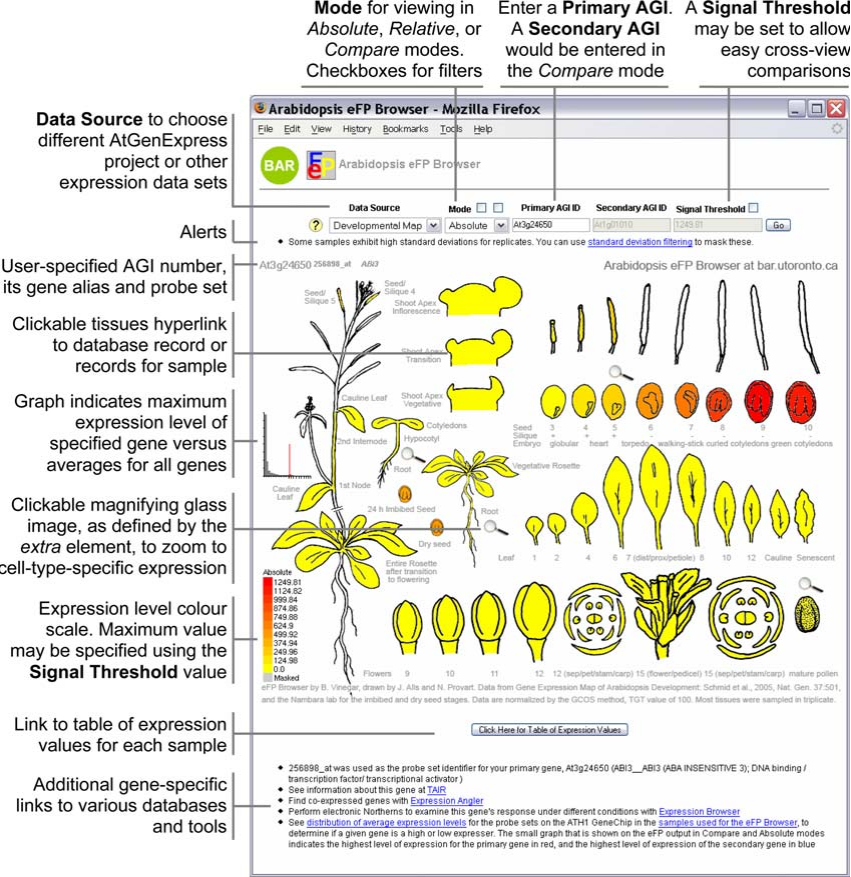
Expression pattern of *ABI3* – *ABSCISIC ACID INSENSITIVE 3*. *ABI3*, At3g24650, is expressed strongly in Stage 8–10 seeds. Features and control options of the Arabidopsis eFP Browser are also shown.

**Figure 3:**
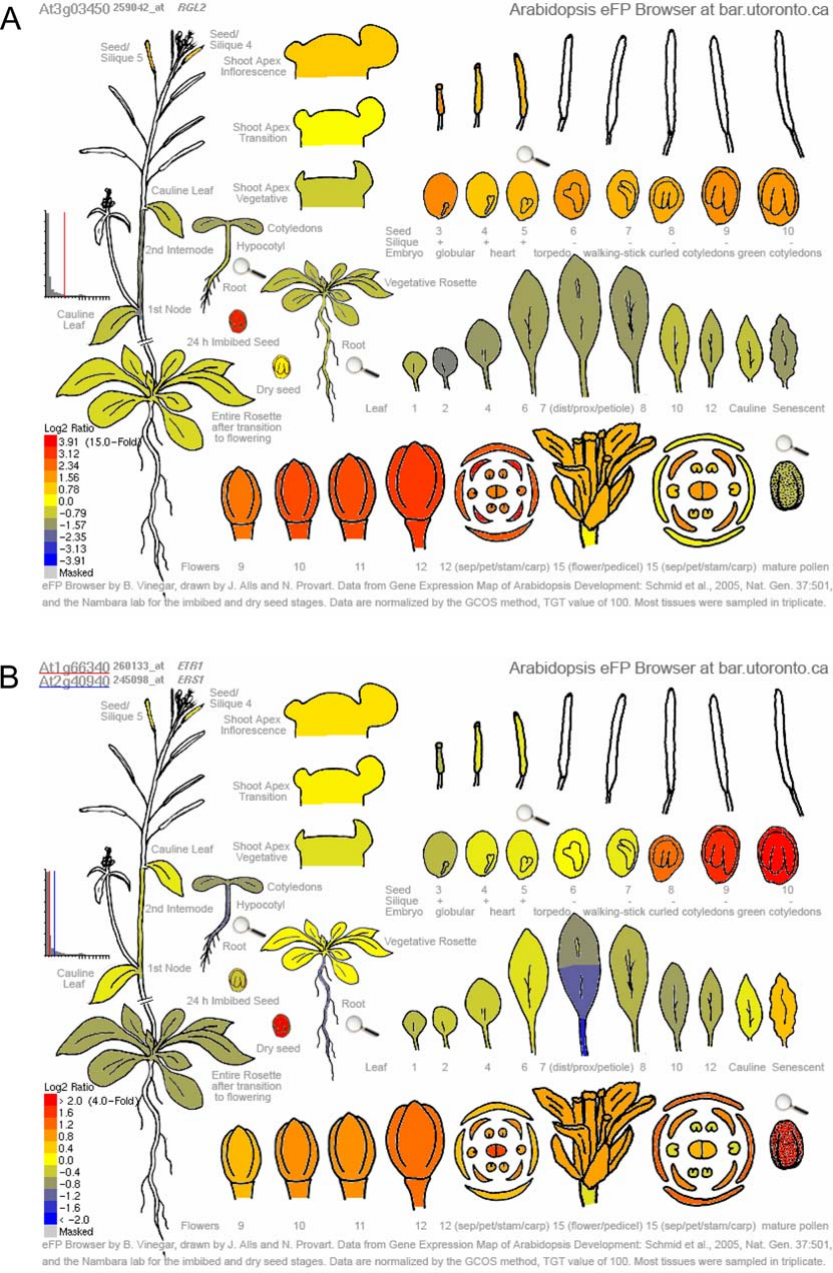
Relative and Compare Modes of the Arabidopsis eFP Browser. A. Expression of *RGL2*, At3g03450, in Relative Expression mode, showing stronger expression relative to its median expression level in imbibed seeds and flower parts, as described in the literature. B. Expression of the ethylene receptors *ERS1*, At2g40940 versus *ETR1*, At1g66340 as visualized in the Compare Mode, showing stronger expression (yellow-green) of *ERS1* in earlier stages of seed development and stronger expression (red) of *ETR1* in later stages of seed development.

For the user, the eFP Browser engine offers three intuitive modes. In “Absolute,” the expression level for a user's gene in each tissue is directly compared to the highest signal recorded for the given gene, with low levels of expression coloured yellow and high levels coloured red. An example **Arabidopsis eFP Browser** output for *ABSCISIC ACID INSENSITIVE 3* (*ABI3*, At3g24650) in the “Absolute” mode is in Figure 2, demonstrating strong expression in Stage 8–10 seeds, where its role in promoting seed dormancy has been documented. In addition, Figure 2 highlights the various output and input features and options of the eFP Browser interface. The “Relative” mode displays the ratio of a tissue's expression level to appropriate control signal – typically the median or mock treatment – for its group, as defined using the <group> and <control> tags in the XML control file, see Figure 1B. (In the case of the Developmental Map and some of the other series we have calculated the median value across all displayed samples for each probe set and loaded these into our database as a separate sample, which is referenced in the XML file with the <control> tag. In other cases, the appropriate untreated control data set value is used to calculate the relative value for the samples within a specified <group>). The output has tissues coloured with expression levels above the control signal value between yellow and red, and expression levels below the control signal value between yellow and blue. An example **Arabidopsis eFP Browser** “relative” output for *RGL2* (At3g03450) is shown in Figure 3A, showing expression levels higher than the median level of expression of *RGL2* in seeds and flowers. Both areas for *RGL2* expression have been described in the literature [13], [14]. The “Compare” mode accepts two gene identifiers as input and compares the primary relative expression levels to the secondary in each tissue, using the same colour scheme described for relative. This is useful for identifying tissues in which one gene is more abundantly expressed relative to another. An example **Arabidopsis eFP Browser** “Compare” output for *ERS1* (At2g40940) compared to *ETR1* (At1g66340) is show in Figure 3B, showing strong levels of expression of *ETR1* relative to *ERS1* in later stage seeds. The single *etr1* mutant exhibits a phenotype in seeds [15].

The eFP Browser was designed to be user-friendly and informative. Hence, several features have been incorporated to increase its value as a resource. The Data Source drop-down options are dynamically generated, based on the XML control files present in the data directory. After the form has been submitted once, the Browser reloads on every change of Data Source, while keeping all the other settings constant. Altering the Mode has a similar effect.

Auxiliary options are provided to adjust the appearance of the produced image, such as imposing a threshold on the displayed values and “greying” out low values or values with a high variability between replicate samples. Checking the Signal Threshold checkbox adjusts the colours by scaling values to the entered threshold instead of the maximum; all values above the positive or negative value of this threshold are displayed as the extreme of the colour scale, i.e. in red or blue. When the first Mode checkbox is filled in the absolute mode, tissues whose standard deviation is greater than 50% of the average value for that tissue are coloured grey to mask them. Some individual gene expression levels for replicate samples – especially those generated by laser-capture microdissection – exhibit a high degree of variability between replicates. The grey effect here alerts the user to this fact. When the second Mode checkbox is filled in the Relative mode, the Browser automatically colours grey all samples where the values used for the ratio calculation are less than 20 expression units, the background level for the AtGenExpress data sets. The grey effect in this case is useful for allowing the user to ignore values that may appear significantly higher relative to their control but are actually not likely biologically meaningful due to their very low absolute expression levels. If filtering or thresholding is not selected, the user is alerted to the fact that filtering or thresholding is possible but only in applicable cases, e.g. if the scale maximum has changed between views or if the replicate values for a given sample in a view exhibit high variation.

Only tissues that have been coloured in the input graphic and indexed with that specific colour in the XML control file will be subject to colour replacement by the eFP Browser engine. In some cases it is instructive to the user to provide additional pictographic information, such as in the case of the Developmental Map in the **Arabidopsis eFP Browser**: only the seeds from later stage siliques were collected for analysis, and not the siliques themselves. It is useful for the biologist, however, to be aware of the appropriate stage of siliques from which the seeds came – this can be achieved by including a sketch without colouration on the input file, in addition to providing text to this effect below the illustration.

In order to decrease the number of errors thrown by the eFP Browser, a number of checks have been added to verify the input. To begin, the form is prevented from being submitted with an improperly formed or absent AGI IDs (or RefSeq IDs in the case of the **Mouse eFP Browser**). If an non-existent AGI ID is entered, an error warning is returned. Furthermore, the Browser reverts to the “No Threshold” setting when the one entered is not appropriate.

After submission, the resultant image illustrates the maximal expression level of the gene or genes of interest on a small representative graph of the distribution of average expression levels for each of ∼22814 genes in the given data set. The distribution is similar for all AtGenExpress data sets, with the exception of the “Development RMA” data set, which was normalized using the RMA method [16] and not the Affymetrix MAS5.0/GCOS method with a target value of 100. This feature allows the user to determine whether a given gene of interest is a “high” or “low” expresser, relative to the average level of expression of all of the genes in a selected data set.

If the user clicks on a tissue, the Browser will direct the user to a relevant experiment link, as specified in the XML control file. In the case of the **Arabidopsis eFP Browser** we link out to the experimental description at NASCArrays [1]. As well, on mouse-over, the tissue's name and expression level – absolute or relative, along with the fold-change or standard deviation – is displayed. A similar feature allows a developer to embedded URLs – either with or without the parameters passed to the eFP Browser – within the image map of the output. An example of such embedded URLs with parameters is seen in Figure 2 in the form of the small magnifying glasses. Clicking on these allows the user to “zoom in” to a tissue-specific data set. These types of embedded URLs have the same effect as changing the Data Source manually to “Tissue Specific”.

A link is provided underneath the image to direct the user to a temporary page listing all the expression values, fold-changes or standard deviation values, and samples names. Also located on the bottom of the page are a variety of links to information on the gene(s), other BAR tools [10], and the XML source file. Lastly, helpful instructions and detailed average expression graphs are available on a click of the question mark link or the miniature distribution graphs on the final image.

### Data Sources and Examples

The eFP Browser was designed with the intention that it can be easily employed on any Data Source provided it is in the correct format. As long as a given data set is present in one of our databases, a user could add another data source by simply supplying three files: an accurately formatted XML file similar to that shown in Figure 1B, and an image in both Portable Network Graphics (.png) and Targa (.tga) format to embody the experiment(s). Further information on adding views to the eFP Browser is available in the Download, Upload and Linking section. Note that the source code is also available for local installation atop an existing database of gene expression or other measurements. Someone with a modest amount of Python programming experience should easily be able to adapt the code for local use, which depends on the local database structure.

**Figure 4:**
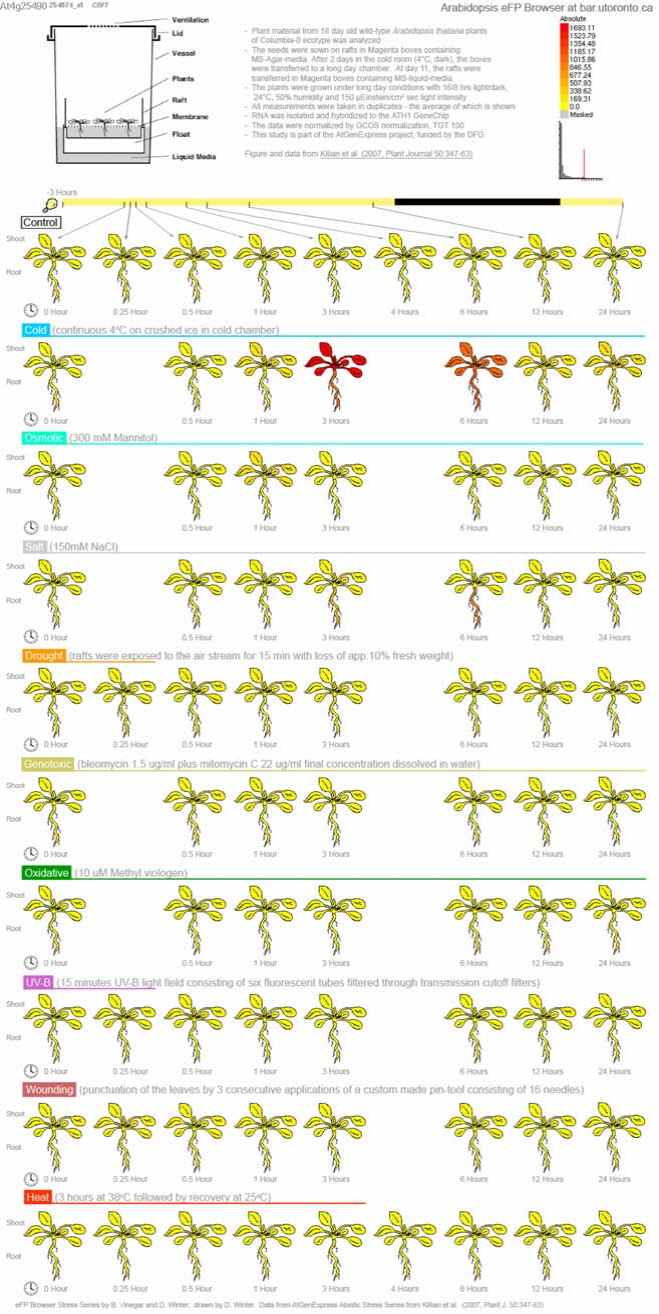
Abiotic stress response for the cold-inducible transcription factor gene *C-BOX BINDING FACTOR 1*, At4g25490, as viewed with the Arabidopsis eFP Browser.

Currently, there are several AtGenExpress series already available to be accessed by the **Arabidopsis eFP Browser**. The first, dubbed Developmental Map, displays a gene expression map of Arabidopsis development [17] plus a dry and germinating seed sample from another AtGenExpress partner. The Abiotic Stress series demonstrates the level expression in the shoot and root of plants under control, cold, osmotic, salt, drought, genotoxic, oxidative, UV-B, wounding, and heat stress conditions [18]. Figure 4 shows the expression of a known cold-inducible transcription factor, CBF1, in response to cold stress [19]. Furthermore, the expression levels of leaves in plants exposed to pathogens including *Botrytis cinerea*, *Pseudomonas syringae*, bacterial- and oomycete-derived elicitors, *Phytophtora infestans,* and *Erysiphe orontii* is the focus of the Biotic Stress Series. Whole seedlings and seeds treated with typical plant hormones or chemicals, such as hormone inhibitors, are presented in the Hormone and Chemical Series, respectively. These data sets were produced by members of the AtGenExpress Consortium.

**Figure 5:**
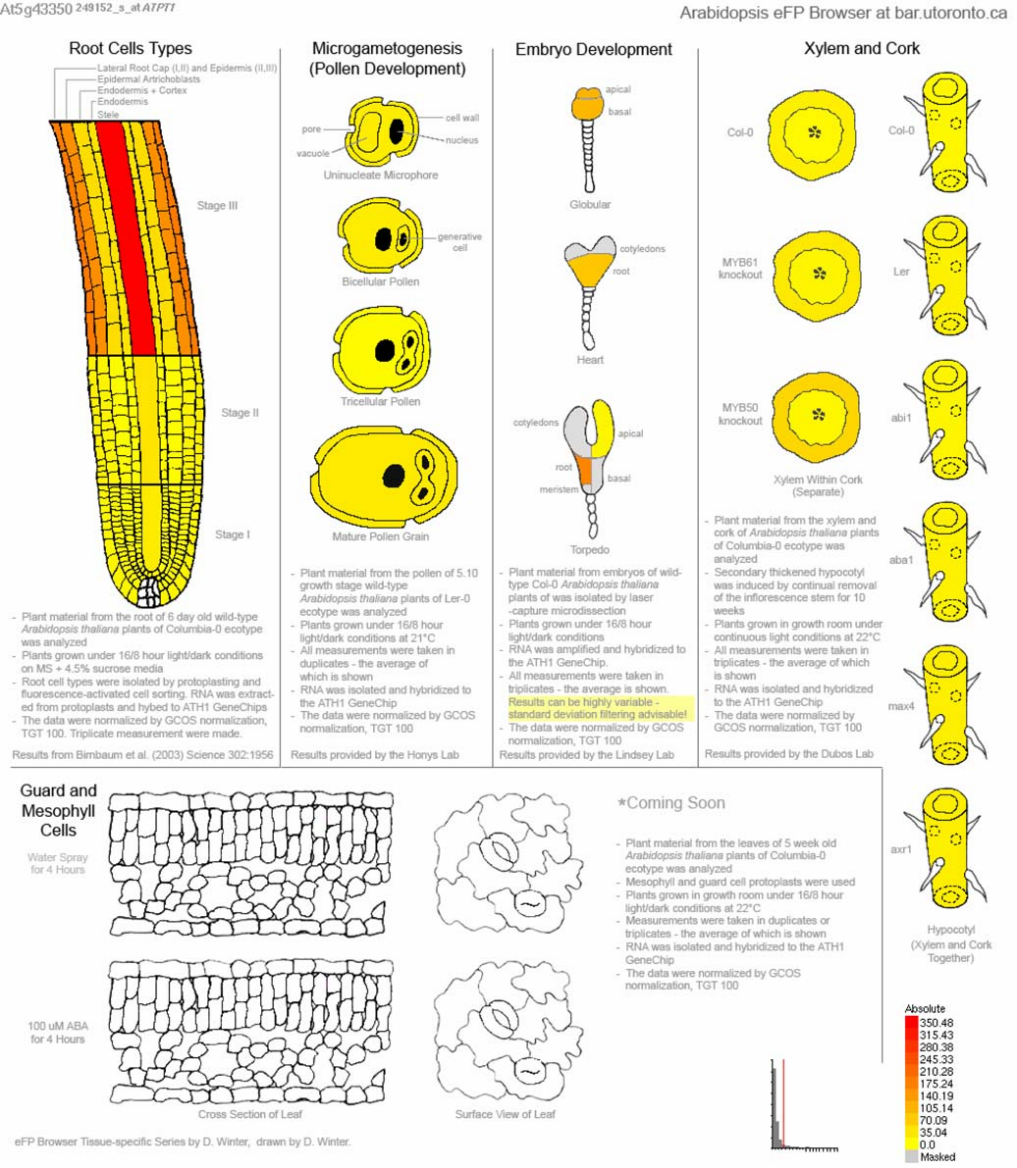
Tissue-specific expression patterns for At5g43350, *AtPT1/Pht1;1*. This gene encodes an inorganic phosphate transporter known to be strongly expressed in the roots. Tissues where values from replicate samples exhibit a high standard deviation are coloured grey – the standard deviation filtering feature of the Arabidopsis eFP Browser was activated during output generation.

In addition, the final data source provides an eclectic collection of tissue-specific samples from a number of independent sources. Links from the Developmental Map and the scroll-down menu allow the user to zoom in on root layers [20], embryogenesis [21], microgametogenesis [22], secondary thickened hypocotyls, and other tissue types as they become available, as shown for *AtPT1*/*Pht1;1*, an inorganic phosphate transporter involved in phosphate uptake in the roots, in Figure 5 [23].

**Figure 6:**
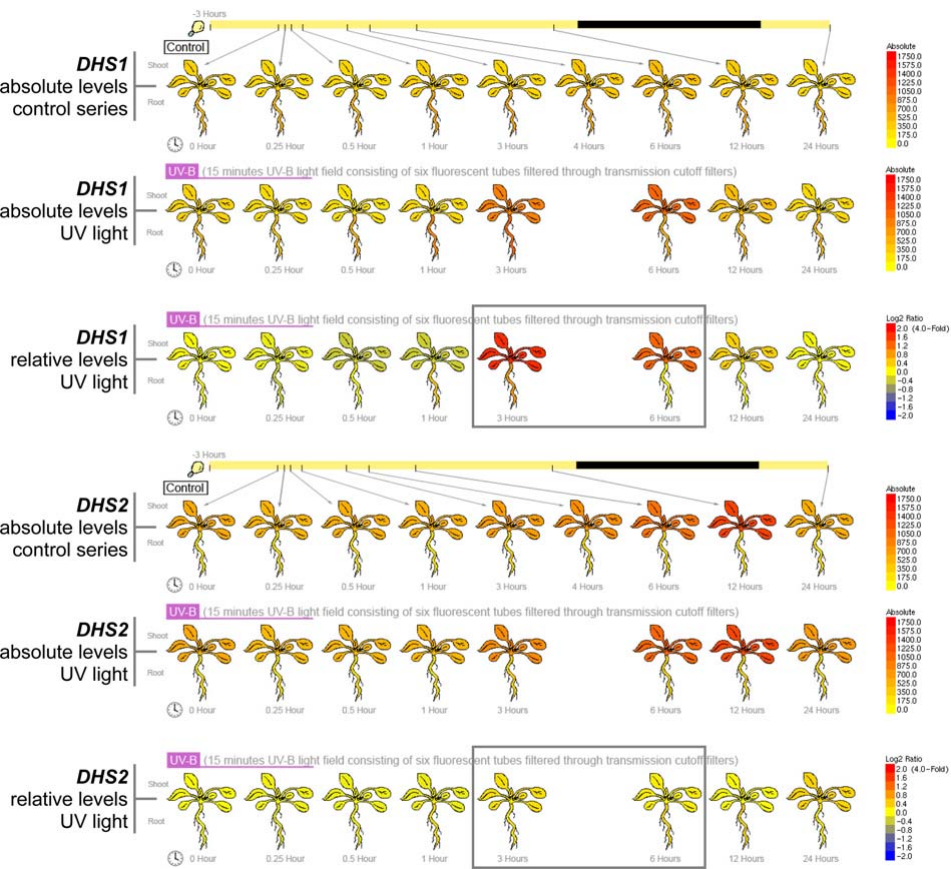
Absolute and relative expression patterns of expression for *DHS1*, At4g39980, and *DHS2*, At4g33510. *DHS1* is strongly induced by UV light treatment, while *DHS2* is diurnally responsive but not UV-inducible *per se*, see boxed timepoints.

**Figure 7:**
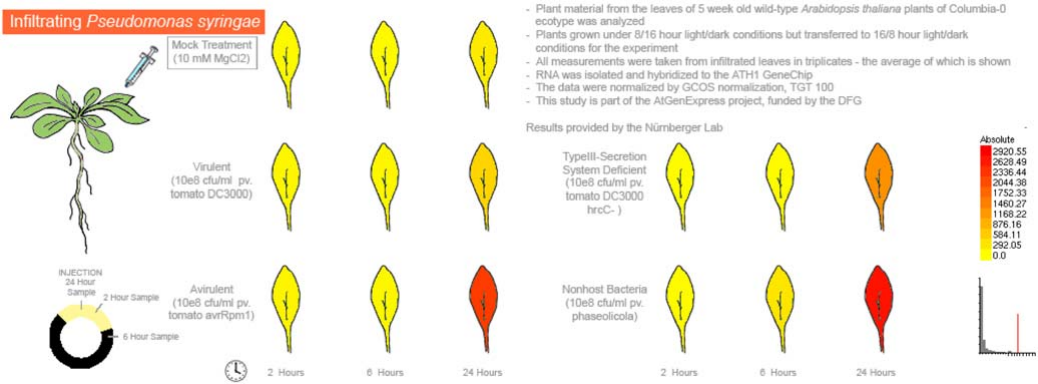
A section of the Arabidopsis eFP Browser Biotic Stress output. Induction of a pathogen-inducible gene, *PR1*, At2g14610, upon *Pseudomonas syringae* attack, is clearly evident 24 hours after inoculation.

We believe that the **Arabidopsis eFP Browser** provides a convenient overview of gene response for these experiments as well as an improved understanding of the experimental set-up for the data set. Light bars and circular illumination regime indicators show the timing of treatment and sampling in the day (Figures 4, 6 and 7). For instance, plants grown for the Abiotic Stress series were grown under a day-night light cycle (16h light), imposing in some cases a diurnal pattern of gene expression response on top of any stress response. This is demonstrated in the expression patterns of two 3-deoxy-D-arabino-heptulosonate 7-phosphate synthase (DHS, EC 2.5.1.54) isoforms. DHS catalyzes the first step in the shikimate pathway for chorismate synthesis, an important precursor for UV protective pigments, among many other compounds. In the “Absolute” mode both are seemingly UV-B inducible, however, the “Relative” mode (Figure 6) makes it clear that *DHS2* is under diurnal control, while *DHS1* is UV-B inducible. Also, for the Abiotic Series, some stresses were applied continuously over 24h, while others were temporarily applied. This is indicated in the eFP Browser output by lines extending for the period of application of the stress, also indicated in Figures 4 and 6.

Moreover, other details of the experiments are integrated in text boxes within the images. Among the information is the number of replicates, age of plants, genetic background, growing conditions, method of microarray data normalization, and the lab responsible. The goal is to give the user an accurate impression of the experimental setup, without having to wade through many hyperlinks to find the information.

### A “Cell eFP Browser” for Arabidopsis

**Figure 8:**
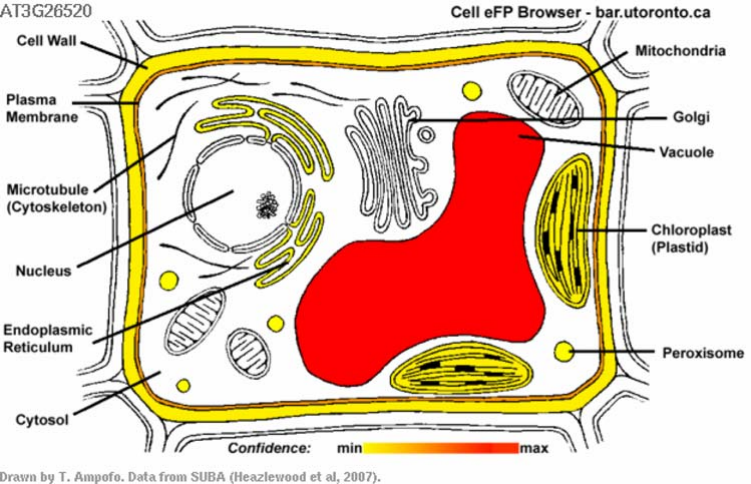
An exemplary Arabidopsis Cell eFP Browser output image. The subcellular localisation pattern of At3g26520, encoding Tonoplast Intrinsic Protein 2, false coloured according to degree of support for a given location based on both measured and predicted subcellular localizations from the SUBA database.

The SUBA database [11] contains information on the computationally predicted and experimentally documented subcellular localization of many Arabidopsis proteins. As an example of displaying discretized data in pictographic format, we have developed the **Cell eFP Browser** for showing a protein's predicted and documented subcellular localizations. We apply the formula indicated in the Material and Methods section to generate a confidence score for each distinct subcellular compartment or region. The higher the confidence score for a given subcellular compartment, the more intense the red colour in the **Cell eFP Browser** output. An exemplary output from the **Cell eFP Browser** for a vacuole-targeted protein, TONOPLAST INTRINSIC PROTEIN 2 (TIP2, At3g26520) is shown in Figure 8.

### An “electronic fluorescent Mouse”

**Figure 9:**
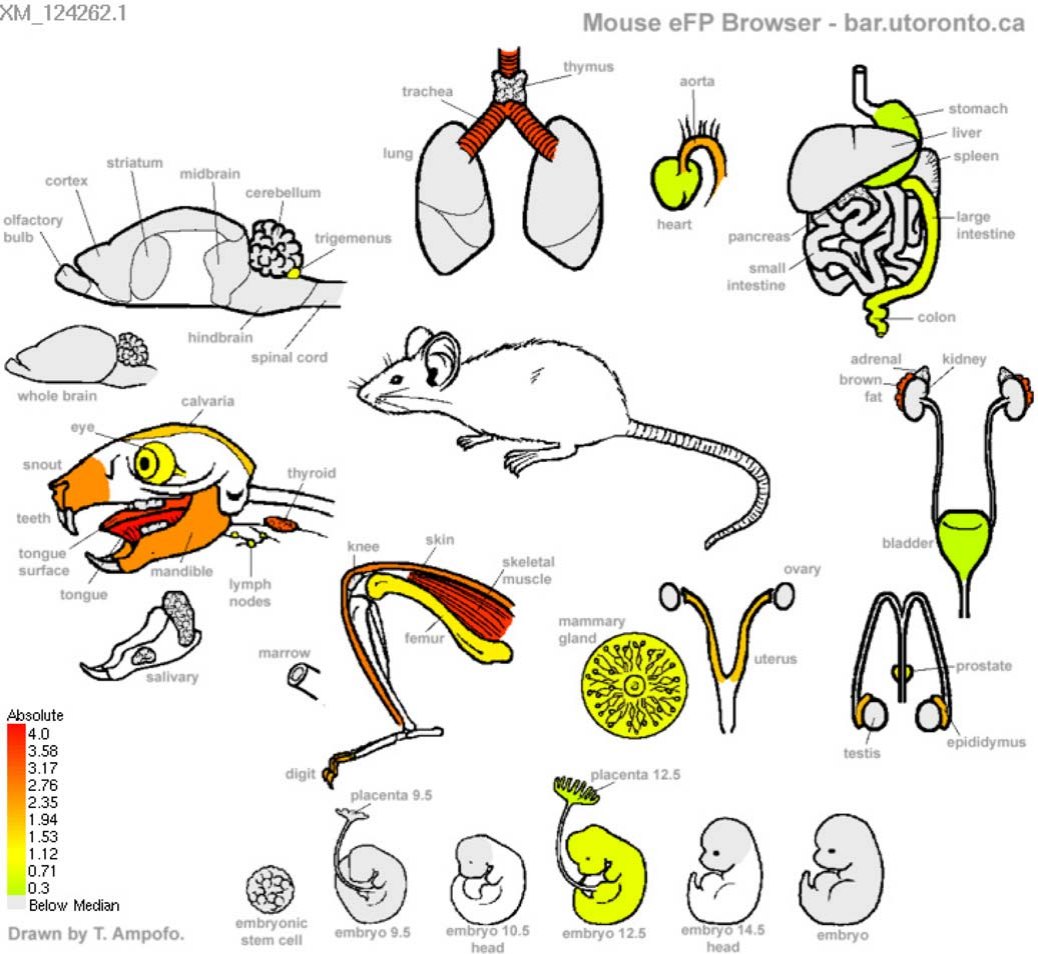
An exemplary Mouse eFP Browser output image. The expression level for myosin (XM_126201.1) is strongest in skeletal muscle and a couple of other areas, such as the trachea, as denoted by the strong red colouration there.

To illustrate the utility of the eFP Browser engine for displaying expression data from 55 tissues in mouse, we have also developed a **Mouse eFP Browser**, based on a data set generated by the Hughes laboratory [12]. We have used the arcsinh-transformed, averaged, median-subtracted and negative-values-zeroed data set from their analysis and, as such, only offer the “Absolute” option for viewing. Figure 9 shows the **Mouse eFP Browser** output for a muscle-specific protein, beta tropomyosin (Tpm2, XM_124262.1).

### Download, Upload and Linking

The **Arabidopsis eFP Browser** for exploring Arabidopsis microarray data encompassing more than 1000 microarray data sets produced by the AtGenExpress Consortium and from other labs is freely available to anyone with a web-browser and an internet connection at http://www.bar.utoronto.ca/, as is the **Cell eFP Browser** and the **Mouse eFP Browser**. Information on obtaining the source code under the Open Source GPL and installation instructions, as well as information on uploading specific data sets from Arabidopsis for public exploration, may be obtained from http://www.bar.utoronto.ca/efp/development/. Details on generating dynamic hyperlinks to the eFP Browser are also available on the development homepage.

### Summary

In conclusion, the eFP Browser is a convenient tool for interpreting and visualizing gene expression and other data. Not only is it valuable for its compatibility to existing resources but it has also been loaded with several useful data sets. The various modes and other features allow the user to extract an array of conclusions and/or generate useful hypotheses. We hope that many researchers will be able to use the eFP Browser both to understand particular microarray or other experimental results, as well as to communicate their own findings.

## Materials and Methods

The eFP Browser is implemented in Python and makes use of the Python Imaging Library (PIL) Build 1.1.5 (www.python.org), which we modified to provide an optimized flood pixel replacement function called replaceFill, and other Python modules, as described on the eFP Browser development homepage. The inputs for the eFP Browser are illustrated in Figure 1. A pictographic representation of the sample collection as a Targa-based image is required, as is an XML control file, shown in detail in Figure 1B. Two other inputs are a database of gene identifiers and their appropriate microarray element lookups and annotations, and a database of gene expression values for the given samples. In the case of the **Arabidopsis, Cell** and **Mouse eFP Browsers**, we have mirrored publicly-available microarray data from several sources – described in the Data Sources and subsequent two sections – in our Bio-Array Resource [10]. These inputs are used by the eFP Browser algorithm to generate an output image for a user's gene identifier.

The eFP Browser algorithm itself is programmed in an object-oriented manner. The main program, efpWeb.cgi, is responsible for the creation of the HTML code for the user interface and presentation of the output image. It calls on four modules to complete the task. These modules are 1) efp.py, which performs most of the functions for the generation of the output image, including the parsing of the XML control file, average and standard deviation calculations, fold-change relative to control value calculations, and image map HTML code; 2) efpDb.py, which connects to the gene expression, microarray element and annotation databases, and returns the appropriate values upon being called; 3) efpImg.py, which formulates the actual colour replace calls on the Targa input image; and 4) efpXML.py, which identifies the XML control files that are present in the eFP Browser's data directory. These are displayed to the user in the Data Source drop-down, thus obviating the need to have them hard-coded in the main efpWeb.cgi program.

In the case of the **Cell eFP Browser**, data in the SUBA database indicate the presence of a given protein in a particular subcellular location, either based on computational methods or as molecularly documented by mass spectrometric analysis of subcellular fractions, GFP fusions etc. [11]. We have used a simple heuristic to turn these data into a confidence score for a given gene product's presence in a given subcellular compartment:
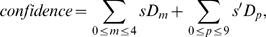



where

*m* = molecular method index of 5 possible methods

*p* = prediction algorithm index of 10 possible algorithms

*s* = weighting for molecular method = 1

*s′* = weighting for prediction algorithm = 0.2

*D* = presence in the subcellular compartment for a given method or algorithm (1 or 0).

The maximum value the confidence score can be for a given compartment is 7 if all methods call a given gene product present in that compartment. While we have arbitrarily given a weighting to prediction algorithm calls for a particular subcellular compartment one fifth that for a molecular method, it would also be possible to incorporate the quality scores for each prediction algorithm instead.
